# Unveiling Telocyte Networks in the Human Superficial Fascia: A New Component of the Hypodermal Interstitial Microenvironment

**DOI:** 10.3390/ijms27209331

**Published:** 2026-10-20

**Authors:** Mirko Manetti, Lucia Petrelli, Alessia Tani, Cesare Tiengo, Irene Rosa, Carla Stecco

**Affiliations:** 1 Section of Anatomy and Histology, Department of Experimental and Clinical Medicine, University of Florence, Largo Brambilla 3, 50134 Florence, Italy; alessia.tani@unifi.it (A.T.); irene.rosa@unifi.it (I.R.); 2 Imaging Platform, Department of Experimental and Clinical Medicine, University of Florence, Viale Gaetano Pieraccini 6, 50139 Florence, Italy; 3 Institute of Human Anatomy, Department of Neurosciences, University of Padova, Via Aristide Gabelli 65, 35121 Padova, Italy; lucia.petrelli@unipd.it (L.P.); carla.stecco@unipd.it (C.S.); 4 Plastic and Reconstructive Surgery Unit, Department of Neurosciences, University of Padova, Via Nicolò Giustiniani 2, 35128 Padova, Italy; cesare.tiengo@unipd.it

**Keywords:** superficial fascia, telocytes, CD34, LGR5, blood vessels, lymphatic vessels, mast cells, autonomic innervation, hypodermis

## Abstract

The human superficial fascia (SF) is a dynamic layer within the hypodermis, increasingly recognized for its role in tissue repair, lymphatic drainage, and fascial dysfunctions. Yet the complete cellular landscape of SF remains largely uncharacterized. Telocytes (TCs) are specialized interstitial cells defined by long, moniliform telopodes, serving as “connecting cells” in various organs. This study provides the first identification of TCs in human SF using immunohistochemistry, confocal immunofluorescence, and transmission electron microscopy (TEM). CD34+ TCs were widespread across both adipose-rich and fibrous compartments of human SF. Confocal microscopy identified extensive three-dimensional networks of CD34+/PDGFRα+/LGR5+/CD31–/α-SMA–/c-kit– TCs, closely associated with CD34+/CD31+ microvessels and TH+ autonomic nerve fibers. Notably, TCs established strategic spatial relationships with D2-40+ lymphatics and c-kit+ mast cells, raising the possibility of a TC–mast cell functional tandem in interstitial fluid homeostasis. Furthermore, the expression of the stemness marker LGR5 positions TCs as an integral component of perivascular stem cell niches. TEM confirmed the presence of TCs ultrastructurally distinguished by characteristic telopodes. In conclusion, our findings suggest that TCs may act as important regulators of SF integrity and homeostasis. Their identification provides novel insights into fascial anatomy while uncovering potential therapeutic targets for conditions such as lymphedema and impaired wound healing.

## 1. Introduction

The human fascial system consists of a dynamic and highly complex three-dimensional (3D) continuum of soft, collagen-containing connective tissues that surround and interconnect muscles, bones, nerves, blood vessels, and viscera, while simultaneously transmitting mechanical forces and adapting to constantly changing loading conditions [1]. Within this framework, the superficial fascia (SF) represents a distinct fibrous layer located in the middle of the hypodermis, separating the superficial adipose tissue from the deep adipose tissue [2]. Once overlooked and considered a mere passive structural membrane, the SF is now recognized as a vital, mechanically active structure endowed with a rich vascularization, extensive autonomic innervation, and a lymphatic plexus [2,3,4,5,6]. These features highlight its active role in thermoregulation, lymphatic drainage, and the maintenance of a smooth gliding interface between the skin and the underlying musculoskeletal system [2].

From a histological perspective, the SF consists of both adipose-rich and fibrous compartments, featuring interwoven collagen bundles interspersed among an abundant network of elastic fibers [2,7]. While fibroblasts have traditionally been regarded as the primary resident cell population, recent findings have demonstrated a more complex cellular landscape, highlighting a significant density of mast cells involved in local inflammatory and tissue-healing processes [2,8]. However, the interstitial microenvironment of the SF remains to be fully characterized, particularly regarding cells that might coordinate the sophisticated intercellular signaling required for tissue homeostasis and remodeling.

Telocytes (TCs) are a distinct population of interstitial (stromal) cells that have attracted growing attention since their formal description by Popescu and Faussone-Pellegrini in 2010 [9]. They are characterized by a small cell body and extremely long, thin, and sinuous cytoplasmic processes, termed telopodes [9,10,11,12]. This unique morphology enables TCs to establish extensive 3D networks throughout the interstitium, allowing interactions with multiple neighboring cells over relatively long distances [10,11,12,13]. To date, TCs have been identified in numerous organs, including the heart, lungs, gastrointestinal tract, skin, skeletal muscle, and reproductive organs, suggesting that they represent a ubiquitous component of connective tissues [11,14,15,16,17,18,19,20,21,22,23,24,25,26,27,28,29,30,31,32,33,34,35]. Although their precise biological functions remain incompletely understood, increasing evidence indicates that TCs play a pivotal role in maintaining tissue homeostasis [12,15,18,22,36]. Their strategic localization and complex network of cellular processes allow them to communicate with fibroblasts, endothelial cells, immune cells, peripheral nerves, smooth muscle cells, and stem/progenitor cells through direct contacts and paracrine signaling, including the release of extracellular vesicles [12,24,36,37,38,39]. Consequently, TCs have been implicated in the regulation of extracellular matrix remodeling, angiogenesis, tissue regeneration, immunomodulation, and stem cell niche maintenance [12,18,36,37,40,41,42,43,44,45,46]. Furthermore, several experimental studies suggest that TCs participate in tissue adaptation to mechanical stimuli, acting as coordinators of intercellular communication within mechanically active connective tissues [12,18,22,47,48,49,50,51].

Hence, the fascial system represents a particularly relevant environment in which to investigate TC biology. Indeed, given their recognized role as stromal coordinators in other connective tissues, TCs are attractive candidates for mediating adaptive responses within the fascial system. Despite this strong biological rationale, evidence supporting the presence of TCs in fascial tissue remains remarkably limited [52,53,54]. At present, their occurrence has only been reported in the deep muscular fascia, particularly in the human fascia lata, where cells displaying ultrastructural features consistent with TCs have been identified by transmission electron microscopy (TEM) [52,53,54]. However, these observations relied primarily on morphological criteria and were not supported by a comprehensive immunophenotypic characterization. Notably, the presence and distribution of TCs within the SF have never been investigated.

Establishing whether TCs are genuine constituents of the SF is of considerable biological and clinical interest. Their identification would not only broaden our understanding of fascial cellular composition but could also provide new insights into the mechanisms underlying SF mechanobiology, extracellular matrix remodeling, and tissue repair. Therefore, the aim of the present study is to investigate the presence of TCs in human SF tissue through an integrated morphological and immunophenotypic approach.

## 2. Results

### 2.1. Histological Architecture of the Human Superficial Fascia

**Figure 1:**
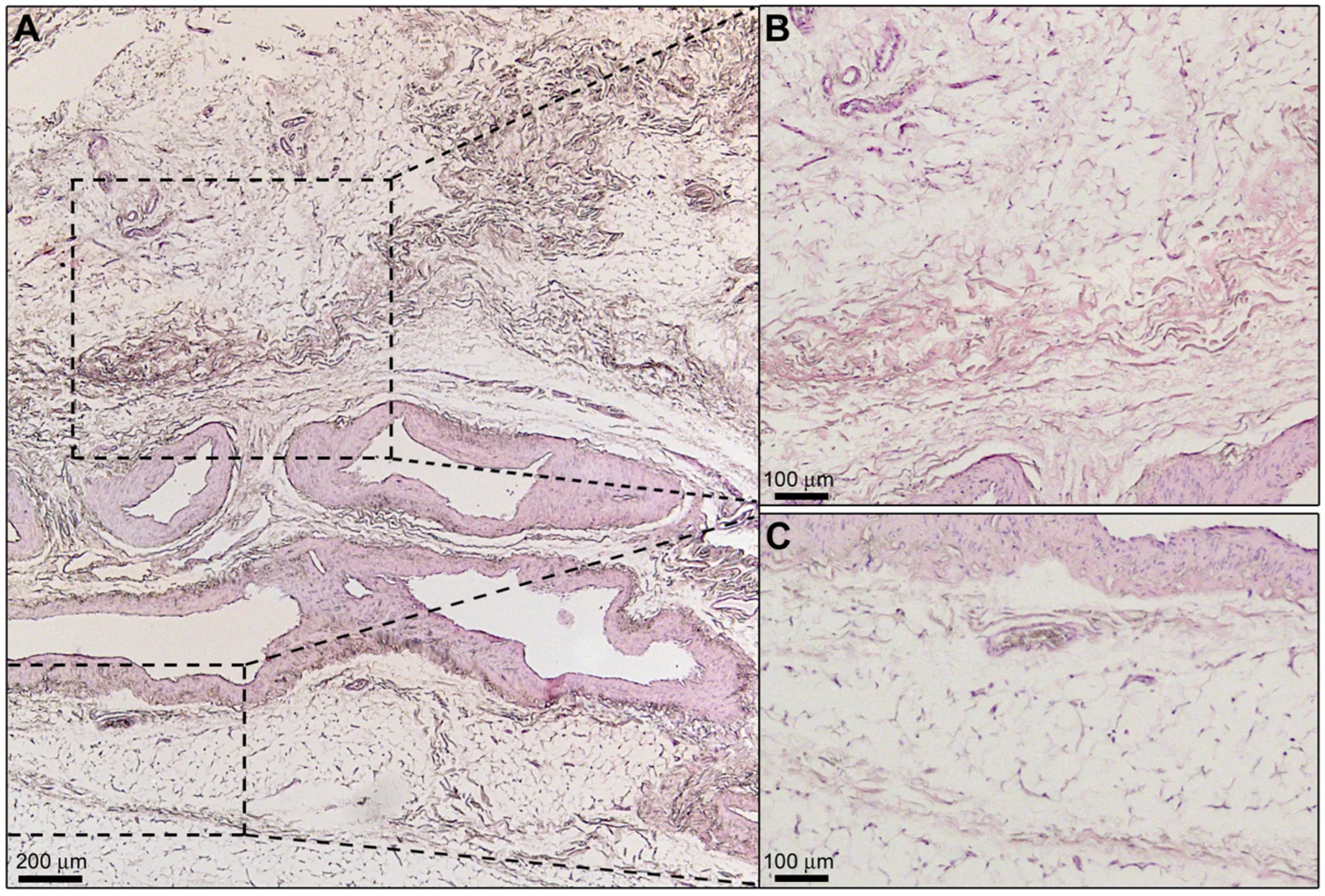
Structural arrangement of the human superficial fascia (SF). (**A**–**C**) Hematoxylin and eosin staining of an SF specimen from the human abdominal wall. (**A**) Overview showing the SF as a highly vascularized and innervated fibrous layer situated within the hypodermis, separating the superficial adipose tissue from the deep adipose tissue. (**B**,**C**) Higher-magnification views of the boxed areas in (**A**) showing the complex pattern of interwoven collagen bundles interspersed with adipose lobules. Photomicrographs are representative of SF tissue sections from three independent biological donors. Scale bars are indicated in each panel.

The histological analysis of human SF tissue sections by hematoxylin and eosin staining confirmed its nature as a distinct fibrous connective tissue layer within the hypodermis, interposed between the superficial and deep adipose tissue (Figure 1A). The SF was characterized by an interwoven network of loosely packed collagen bundles interspersed with adipose cell clusters and numerous vascular and nervous structures, providing a complex 3D scaffold (Figure 1A–C).

### 2.2. Identification and Spatial Distribution of CD34+ Telocytes in the Human Superficial Fascia

**Figure 2:**
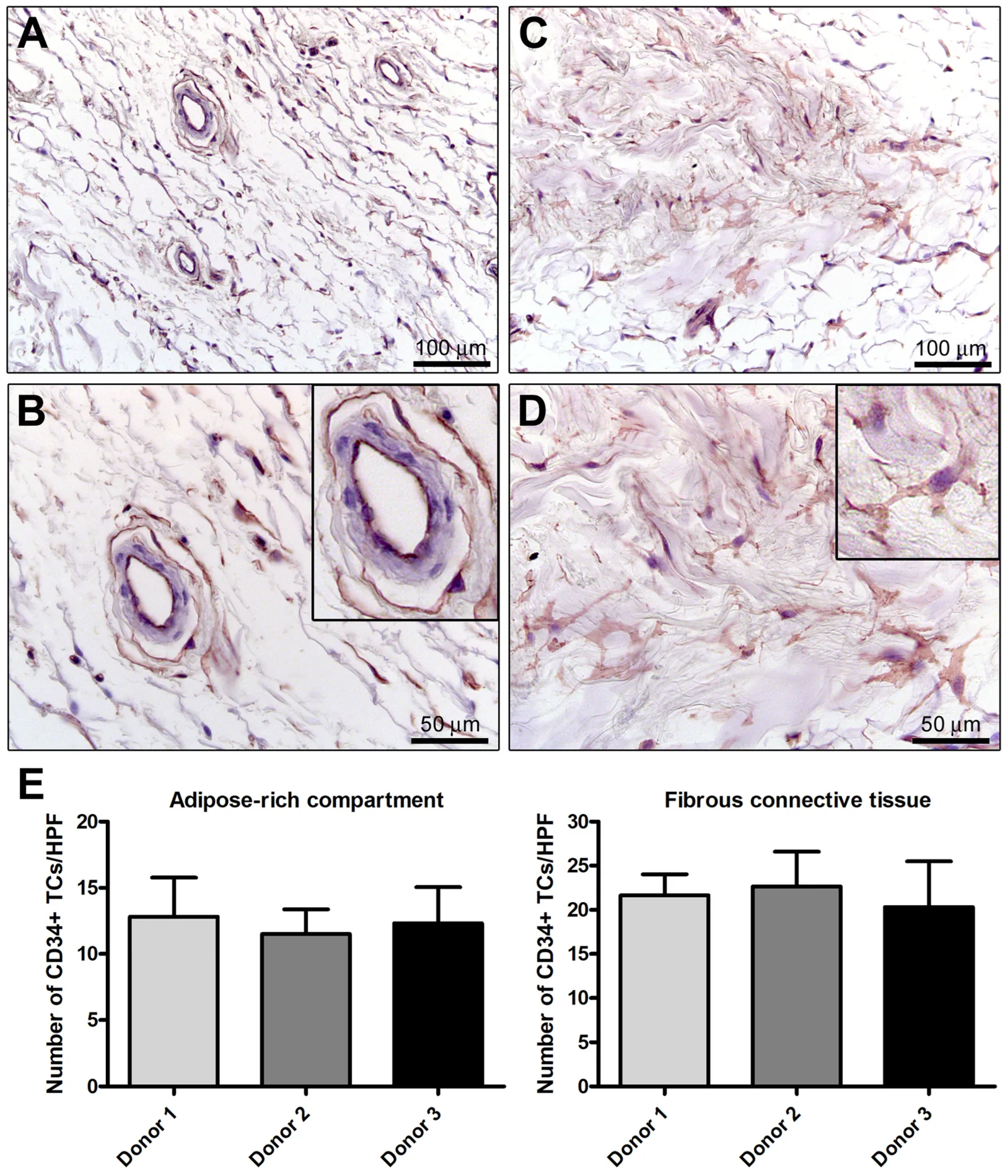
Distribution and morphology of CD34+ telocytes (TCs) in the human superficial fascia (SF). (**A**–**D**) Immunohistochemical staining for CD34 (brownish-red) with hematoxylin nuclear counterstain. (**A**,**B**) Adipose-rich compartment of the SF; (**B**) higher-magnification view of (**A**). The inset in (**B**) shows, at higher magnification, two CD34+ TCs with a spindle- or triangular-shaped cell body and characteristically long and thin processes (telopodes) in a perivascular position within the SF adipose tissue. Endothelial cells of blood vessels also display CD34 positivity (**A**,**B**). (**C**,**D**) Fibrous connective tissue of the SF; (**D**) higher-magnification view of (**C**). The inset in (**D**) illustrates a typical spindle-shaped CD34+ TC with its characteristic processes interspersed among SF collagen bundles. Photomicrographs are representative of SF tissue sections from three independent biological donors. Scale bars are indicated in each panel. (**E**) Bars represent the mean ± SD of the number of CD34+ TCs/high-power field (HPF) in the adipose-rich compartment and the fibrous connective tissue of the SF for each donor (Donor 1, 2, and 3). Quantitative analysis was performed as detailed in Materials and Methods. No significant differences were observed among the three donors (one-way ANOVA with Tukey’s post hoc test).

**Figure 3:**
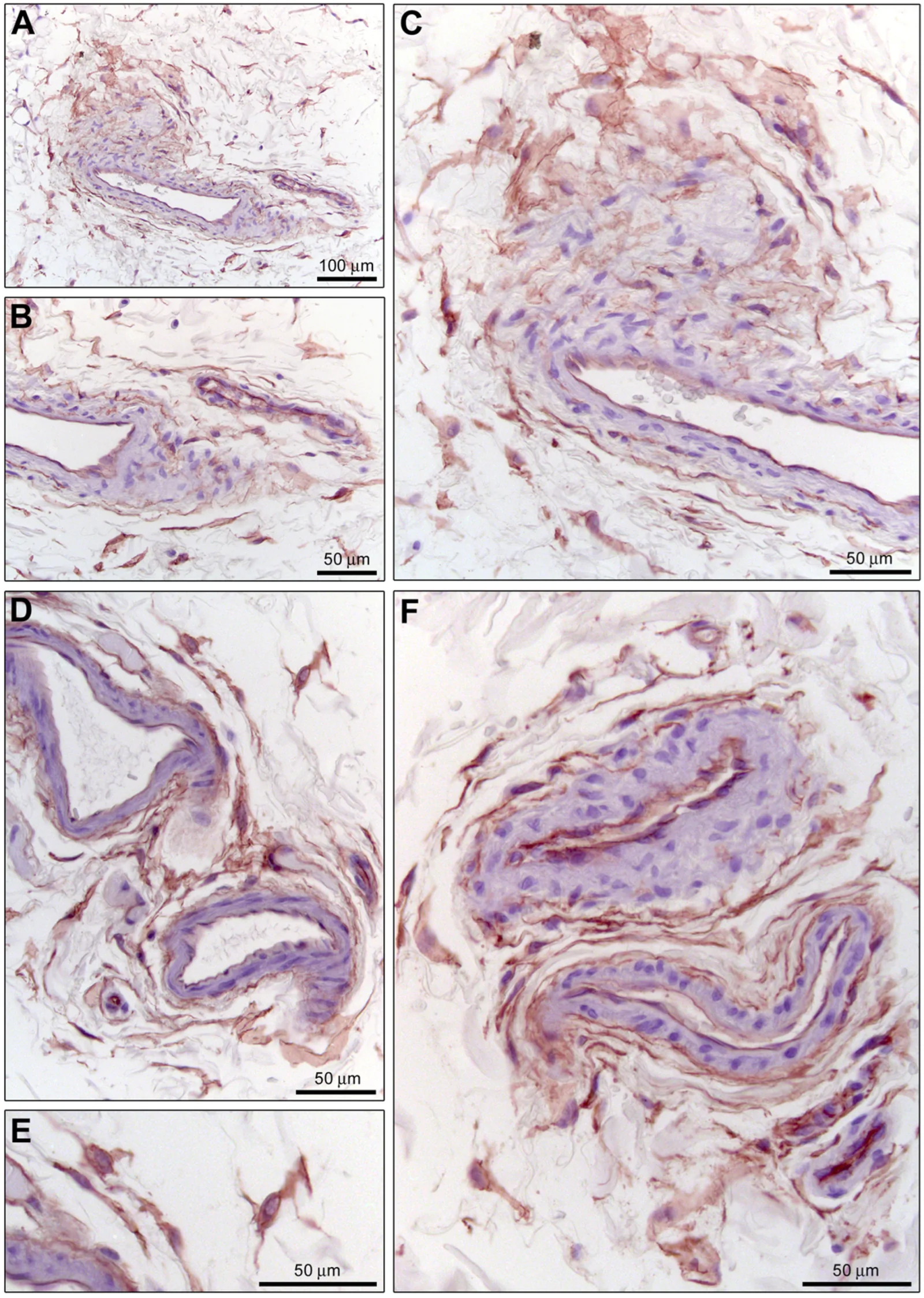
Perivascular networks of CD34+ telocytes (TCs) in the human superficial fascia (SF). (**A**–**F**) Immunohistochemical staining for CD34 (brownish-red) with hematoxylin nuclear counterstain. (**A**–**C**) Extensive network of CD34+ TCs with characteristically long and thin telopodes arranged around a blood vessel; (**B**,**C**) higher-magnification views of (**A**). (**D**–**F**) Further evidence of the peculiar perivascular organization of CD34+ TCs within the SF connective tissue; (**E**) higher-magnification view of (**D**). Note the typical moniliform appearance of telopodes conferred by the alternation of thin segments (podomers) and small dilated portions (podoms) (**E**,**F**). Notably, CD34 immunoreactivity is detectable both in the interstitial TC networks and in the endothelial cells of blood vessels (**A**–**F**). Photomicrographs are representative of SF tissue sections from three independent biological donors. Scale bars are indicated in each panel.

In line with extensive literature [11,12,15,18,22,36,37,41,55], immunohistochemical staining for CD34 served as a primary screening for TCs (Figure 2 and Figure 3). CD34+ stromal cells were ubiquitously distributed throughout the SF, identified in both the adipose-rich compartment and the fibrous connective tissue composed of abundant collagen bundles (Figure 2A–D and Supplementary Figure S1). These cells exhibited the typical morphology of TCs: a small, spindle- or triangular-shaped cell body giving rise to characteristically long, thin, and moniliform processes (telopodes) that followed a sinuous trajectory among adipocytes and collagen bundles (Figure 2B,D). As shown in Figure 2E, quantitative analysis did not reveal any statistically significant difference in the number of CD34+ TCs/high-power field (HPF) among samples from the three donors, in both the adipose-rich compartment and the fibrous connective tissue of the SF. Notably, CD34+ TCs were frequently observed in a prominent perivascular position, forming extensive cellular networks around blood vessels, particularly small arteries/arterioles and venules (Figure 3A–F and Supplementary Figure S1). As expected, CD34 immunoreactivity was also detected in the endothelial cells lining the vascular lumen (Figure 2A,B and Figure 3A–F).

### 2.3. Immunophenotypic Differentiation of Superficial Fascial Telocytes from Vascular Endothelial Cells and Smooth Muscle Cells

**Figure 4:**
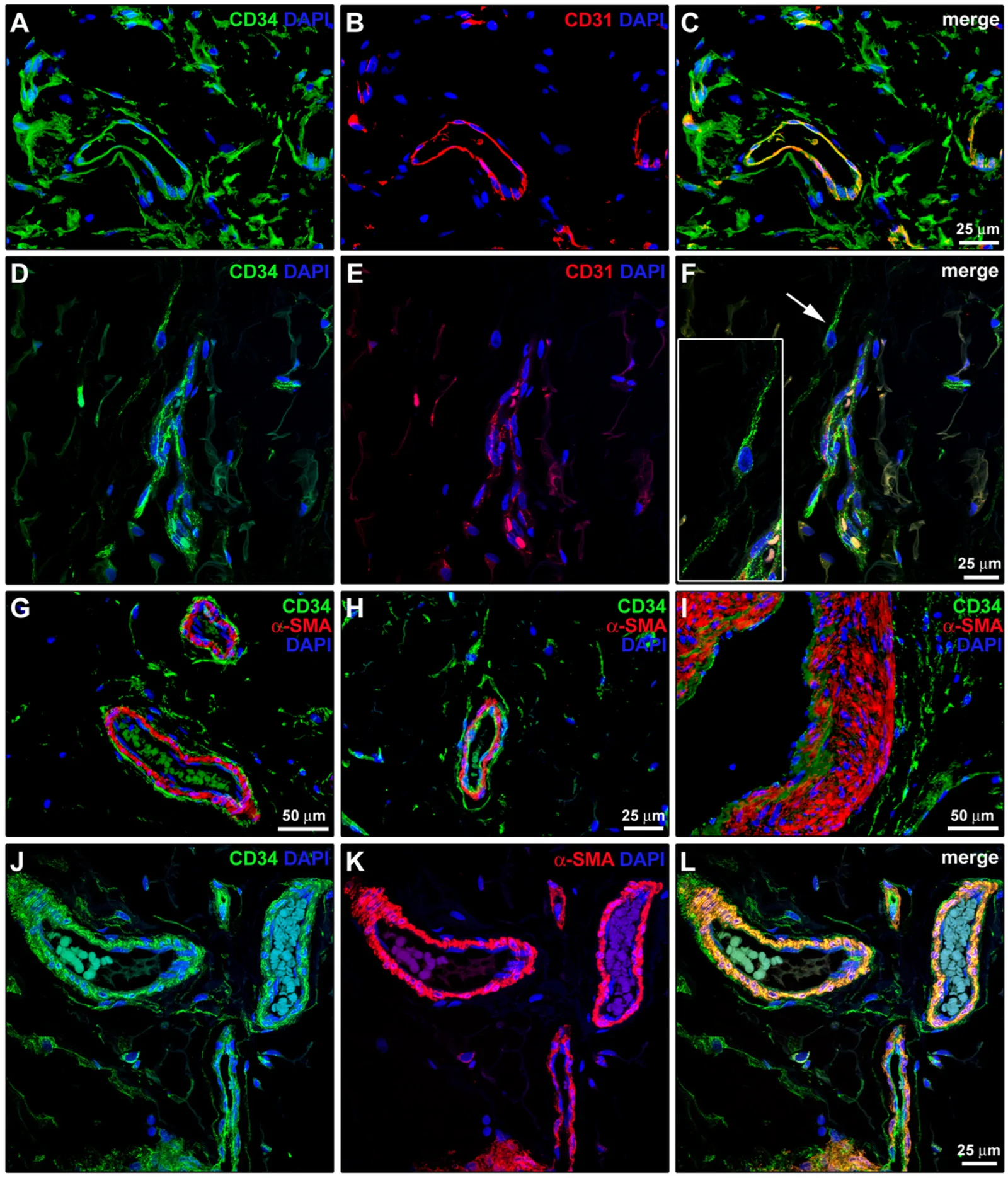
Immunophenotypic distinction and spatial arrangement of CD34+ superficial fascial telocytes (TCs) relative to vascular endothelial cells and smooth muscle cells. (**A**–**C**,**G**–**I**) Representative immunofluorescence images. (**D**–**F**,**J**–**L**) Representative 3D maximum projection confocal laser scanning microscopy images. In all panels, nuclei are counterstained in blue with 4′,6-diamidino-2-phenylindole (DAPI). (**A**–**F**) Double labeling for CD34 (green) and the pan-endothelial marker CD31 (red). Single-channel images for CD34 (**A**,**D**) and CD31 (**B**,**E**), alongside merged images (**C**,**F**), are shown. TCs are identified as CD34+/CD31– interstitial networks surrounding CD34+/CD31+ fascial vessels (**C**,**F**). The inset in (**F**) shows at higher magnification the CD34+/CD31– telocyte indicated by the arrow in the main panel, highlighting its characteristic thin and sinuous telopodes. (**G**–**L**) Double labeling for CD34 (green) and α-smooth muscle actin (α-SMA; red). CD34+ TCs do not express α-SMA and are adventitial cells strategically positioned outside the α-SMA+ vascular smooth muscle cells of fascial small arteries/arterioles and venules (**G**–**I**). Confocal microscopy highlights the intimate TC-vessel relationship, with telopodes forming a dense 3D network that externally envelopes the vascular smooth muscle cell layer (**J**–**L**). Single-channel images for CD34 (**J**) and α-SMA (**K**), alongside the merged image (**L**), are shown. Photomicrographs are representative of superficial fascia tissue sections from three independent biological donors. Scale bars are indicated where applicable.

To accurately distinguish CD34+ TCs from the microvasculature, double immunostaining for CD34 and the pan-endothelial marker CD31/platelet-endothelial cell adhesion molecule-1 (PECAM-1) was performed (Figure 4A–F). This analysis demonstrated that the interstitial networks of TCs were CD34+/CD31–, clearly differentiating them from the adjacent CD34+/CD31+ vascular endothelial cells (Figure 4C,F). Furthermore, double labeling for CD34 and α-smooth muscle actin (α-SMA) revealed that CD34+ TCs do not express α-SMA but are strategically positioned as an adventitial layer immediately outside the α-SMA+ vascular smooth muscle cells of small arteries/arterioles and venules (Figure 4G–L).

### 2.4. Relationship of Superficial Fascial Telocytes with Lymphatic Vessels and Mast Cells

**Figure 5:**
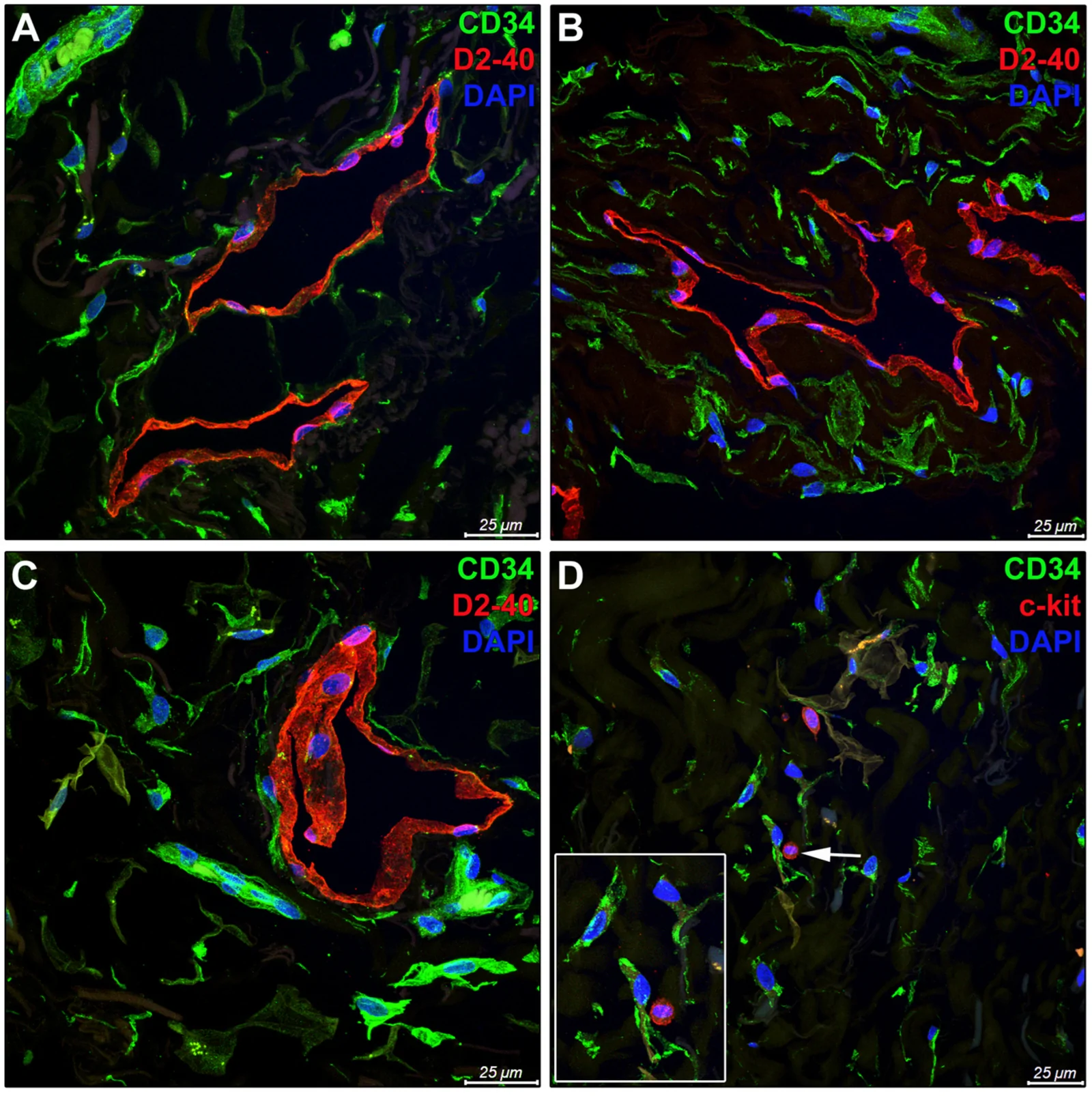
Spatial relationship of telocytes (TCs) with the lymphatic system and mast cells in the human superficial fascia (SF). (**A**–**D**) Representative 3D maximum projection confocal laser scanning microscopy images. In all panels, nuclei are counterstained in blue with 4′,6-diamidino-2-phenylindole (DAPI). (**A**–**C**) Double labeling for CD34 (green) and the lymphatic endothelial marker podoplanin/D2-40 (red). (**A**,**B**) Extensive interstitial networks of CD34+ TCs are distributed around D2-40+ lymphatic capillaries of the SF. (**C**) CD34+ TCs are observed surrounding a D2-40+ lymphatic pre-collector, which is clearly recognizable by the presence of a lymphatic valve. (**D**) Double labeling for CD34 (green) and c-kit/CD117 (red). The SF interstitial network of TCs is characterized by a CD34+/c-kit– immunophenotype, whereas c-kit immunoreactivity is exclusively detectable in oval/round-shaped mast cells. The inset in (**D**) shows at higher magnification a TC close to a c-kit+ mast cell (indicated by the arrow in the main panel). Photomicrographs are representative of SF tissue sections from three independent biological donors. Scale bars are indicated in each panel.

The spatial relationship of TCs with the SF lymphatic system was evaluated by confocal microscopy using double labeling for CD34 and the lymphatic endothelial marker podoplanin/D2-40 (Figure 5A–C). A rich 3D network of CD34+ TCs was identified surrounding D2-40+ lymphatic capillaries (Figure 5A,B) and pre-collectors (Figure 5C). Moreover, as demonstrated by double labeling for CD34 and c-kit/CD117, CD34+ TCs were frequently observed close to c-kit+ mast cells in the SF connective tissue (Figure 5D). Of note, this analysis also demonstrated that TCs in the human SF are immunonegative for c-kit (Figure 5D).

**Figure 6:**
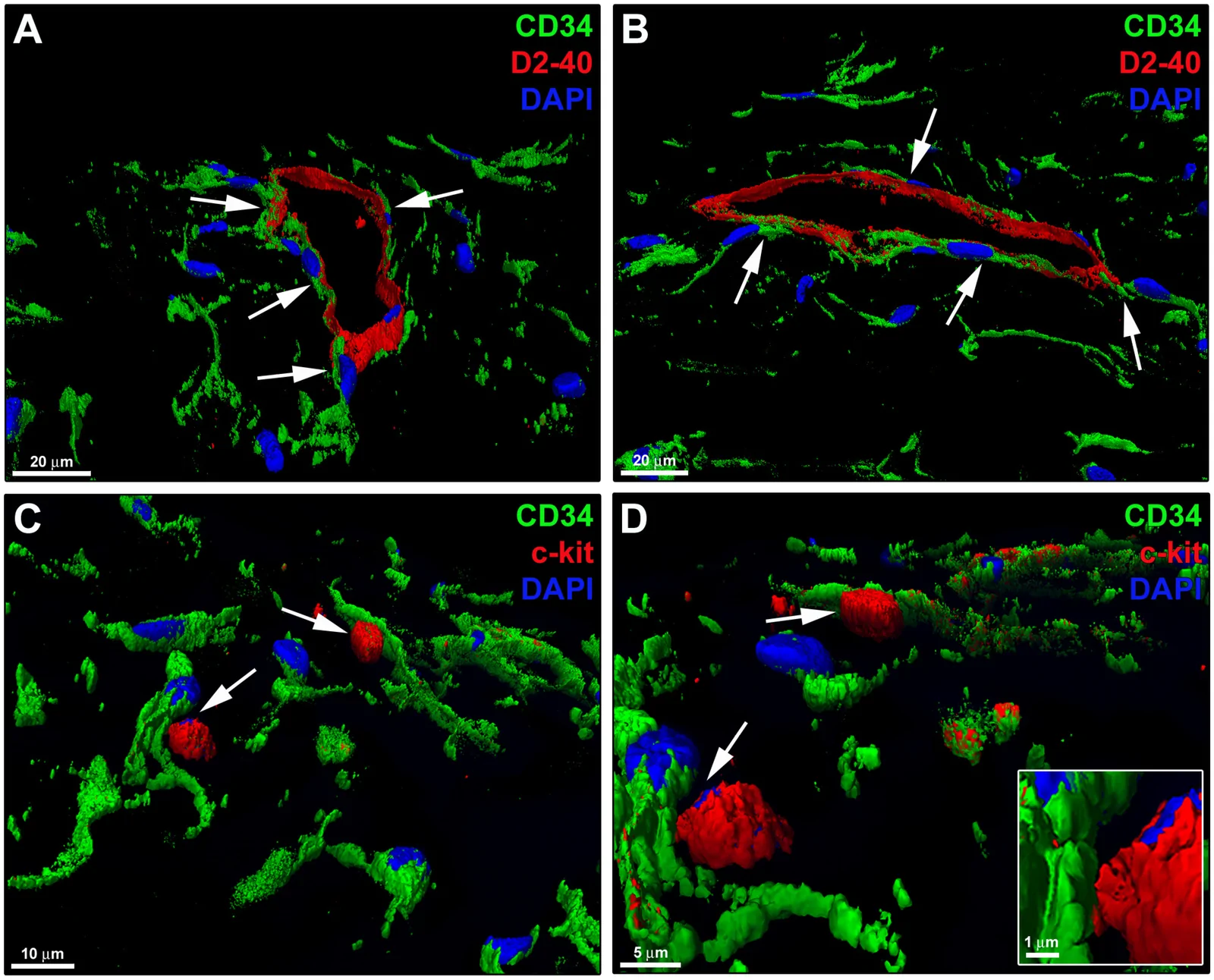
Representative 3D volume renderings of CD34/D2-40 and CD34/c-kit confocal immunofluorescence of human superficial fascia (SF). (**A**,**B**) CD34+ TCs (green) are spatially arranged around D2-40+ endothelial cells of a lymphatic capillary (red), intimately lining their abluminal side (arrows). (**C**,**D**) CD34+ TCs (green) are observed in close spatial proximity to c-kit+ mast cells (red) (arrows). The inset in (**D**) shows a higher-magnification detail of the adjacent plasma membranes of a TC and a mast cell. In all panels, nuclei are counterstained in blue with 4′,6-diamidino-2-phenylindole (DAPI). Images are representative of SF tissue sections from three independent biological donors. Scale bars are indicated in each panel.

To gain further insights into the spatial association between TCs and both lymphatic vessels and mast cells, we performed 3D volume renderings of CD34/D2-40 and CD34/c-kit confocal immunofluorescence (Figure 6). This analysis clearly demonstrated that CD34+ TCs were arranged around lymphatic capillaries, intimately bordering the abluminal side of D2-40+ lymphatic endothelial cells with their cytoplasmic processes (Figure 6A,B). Moreover, the 3D volume rendering highlighted intimate spatial relationships between CD34+ TCs and c-kit+ mast cells (Figure 6C,D).

### 2.5. Expression of PDGFRα and LGR5 Markers in Superficial Fascial Telocytes

**Figure 7:**
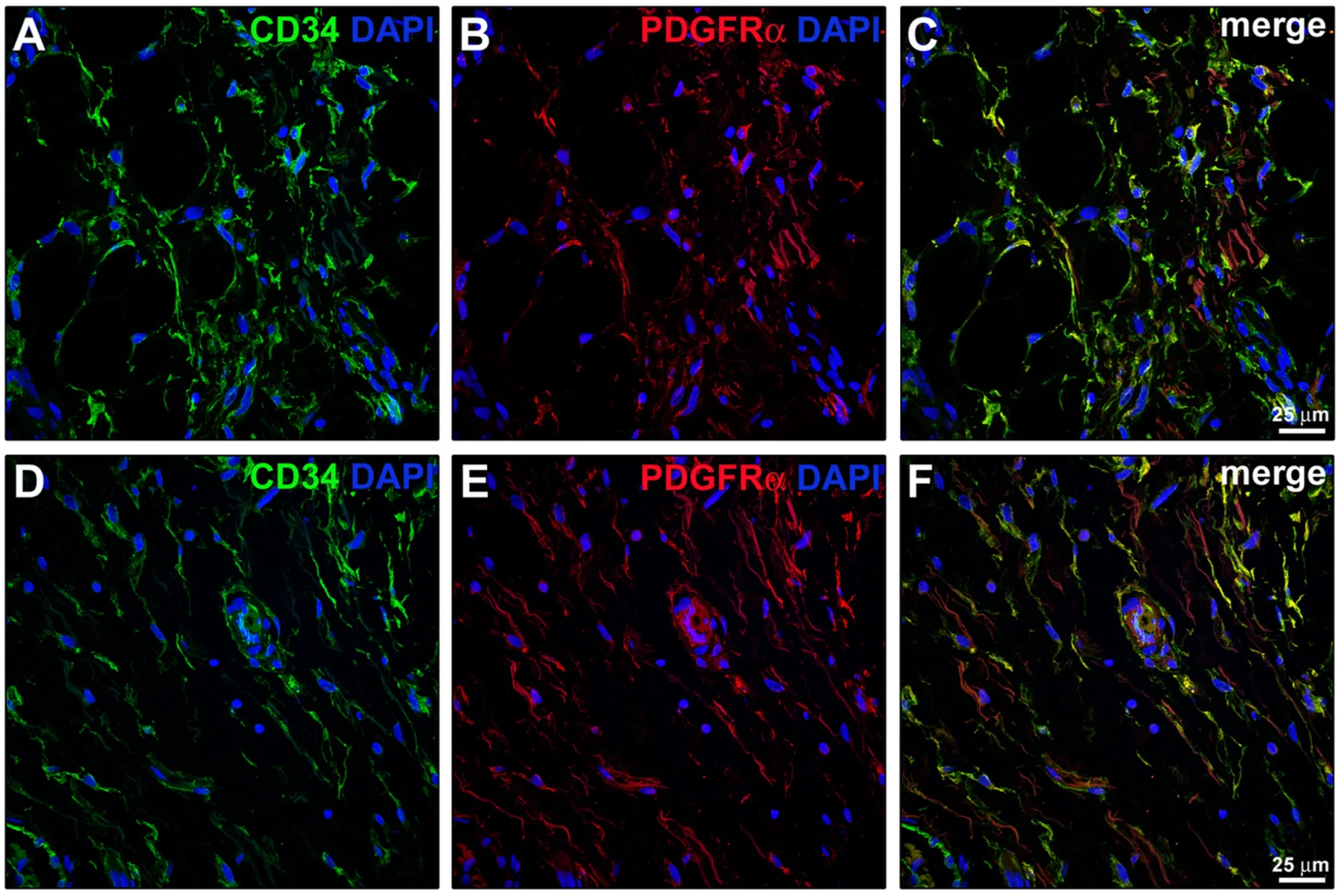
Co-expression of CD34 and platelet-derived growth factor receptor α (PDGFRα) in telocytes (TCs) across different compartments of the human superficial fascia (SF). (**A**–**F**) Representative 3D maximum projection confocal laser scanning microscopy images of SF sections immunostained for CD34 (green) and PDGFRα (red). Single-channel images for CD34 (**A**,**D**) and PDGFRα (**B**,**E**), alongside merged images (**C**,**F**), are shown. In all panels, nuclei are counterstained in blue with 4′,6-diamidino-2-phenylindole (DAPI). (**A**–**C**) Adipose-rich compartment and (**D**–**F**) fibrous compartment of the SF. CD34+ TCs strongly co-express PDGFRα in both fascial compartments, as testified by the orange/yellow staining in the merged images (**C**,**F**). Photomicrographs are representative of SF tissue sections from three independent biological donors. Scale bars are indicated where applicable.

To further characterize the immunophenotype of TCs in the human SF, the expression of platelet-derived growth factor receptor α (PDGFRα) in relation to CD34 was investigated (Figure 7). In both the adipose (Figure 7A–C) and fibrous (Figure 7D–F) compartments of the SF, CD34+ TCs showed a strong co-expression of PDGFRα, a hallmark feature of TCs across various tissues [11,12,36,37,41].

**Figure 8:**
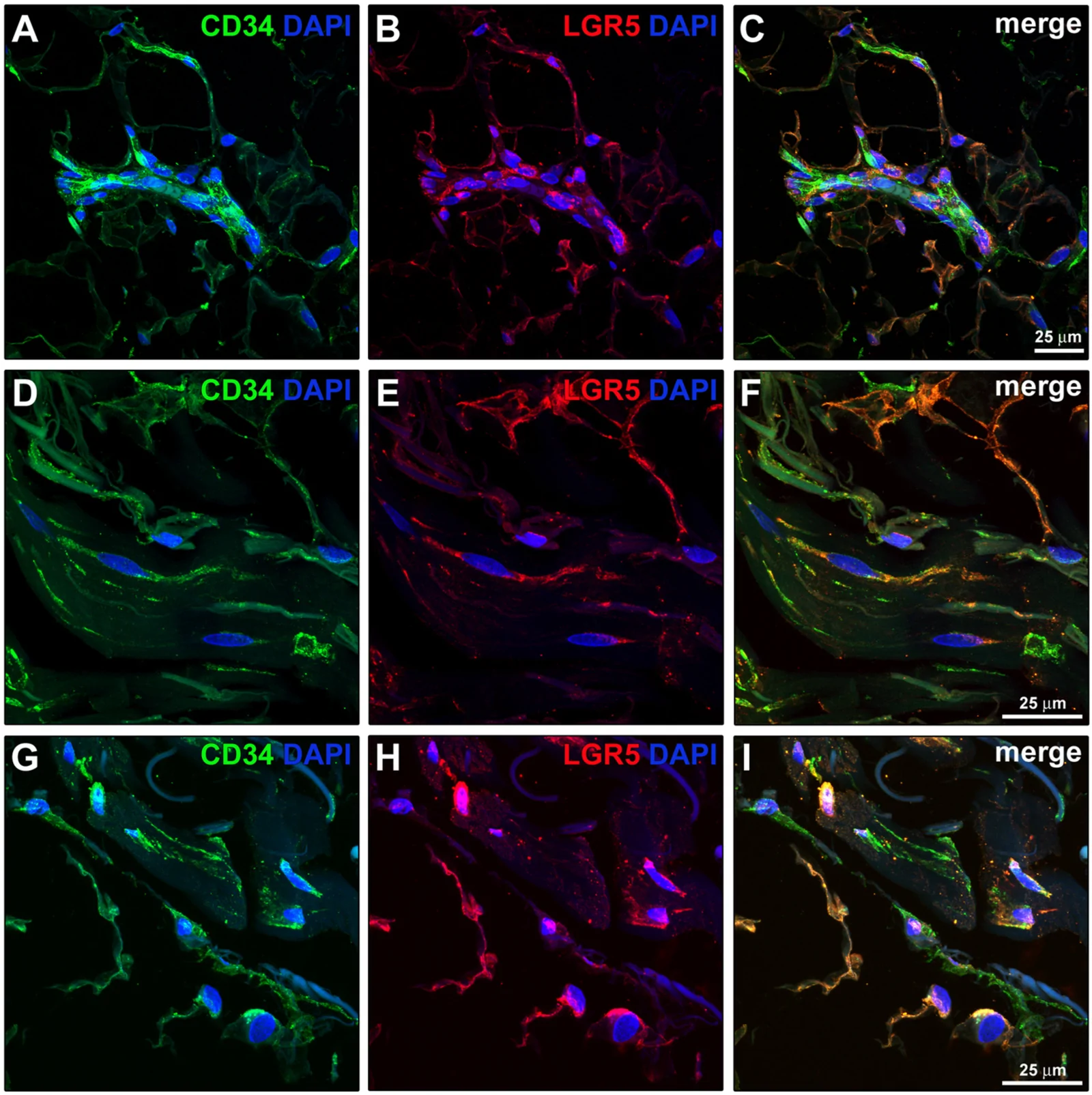
Expression of the stemness marker leucine-rich repeat-containing G-protein-coupled receptor 5 (LGR5) in perivascular telocytes (TCs) of the human superficial fascia (SF). (**A**–**I**) Representative 3D maximum projection confocal laser scanning microscopy images of SF sections immunostained for CD34 (green) and LGR5 (red). In all panels, nuclei are counterstained in blue with 4′,6-diamidino-2-phenylindole (DAPI). Single-channel images for CD34 (**A**,**D**,**G**) and LGR5 (**B**,**E**,**H**), alongside merged images (**C**,**F**,**I**), are shown. CD34+/LGR5+ TCs are strategically positioned around blood vessels, whose endothelial lining is also CD34+. Photomicrographs are representative of SF tissue sections from three independent biological donors. Scale bars are indicated where applicable.

Additionally, double immunostaining for CD34 and leucine-rich repeat-containing G-protein-coupled receptor 5 (LGR5) revealed that perivascular TCs in the human SF express LGR5 (Figure 8A–I), a marker recently associated with TCs in stem cell niches [24,56].

### 2.6. Spatial Association of Superficial Fascial Telocytes with Autonomic Nerve Fibers

**Figure 9:**
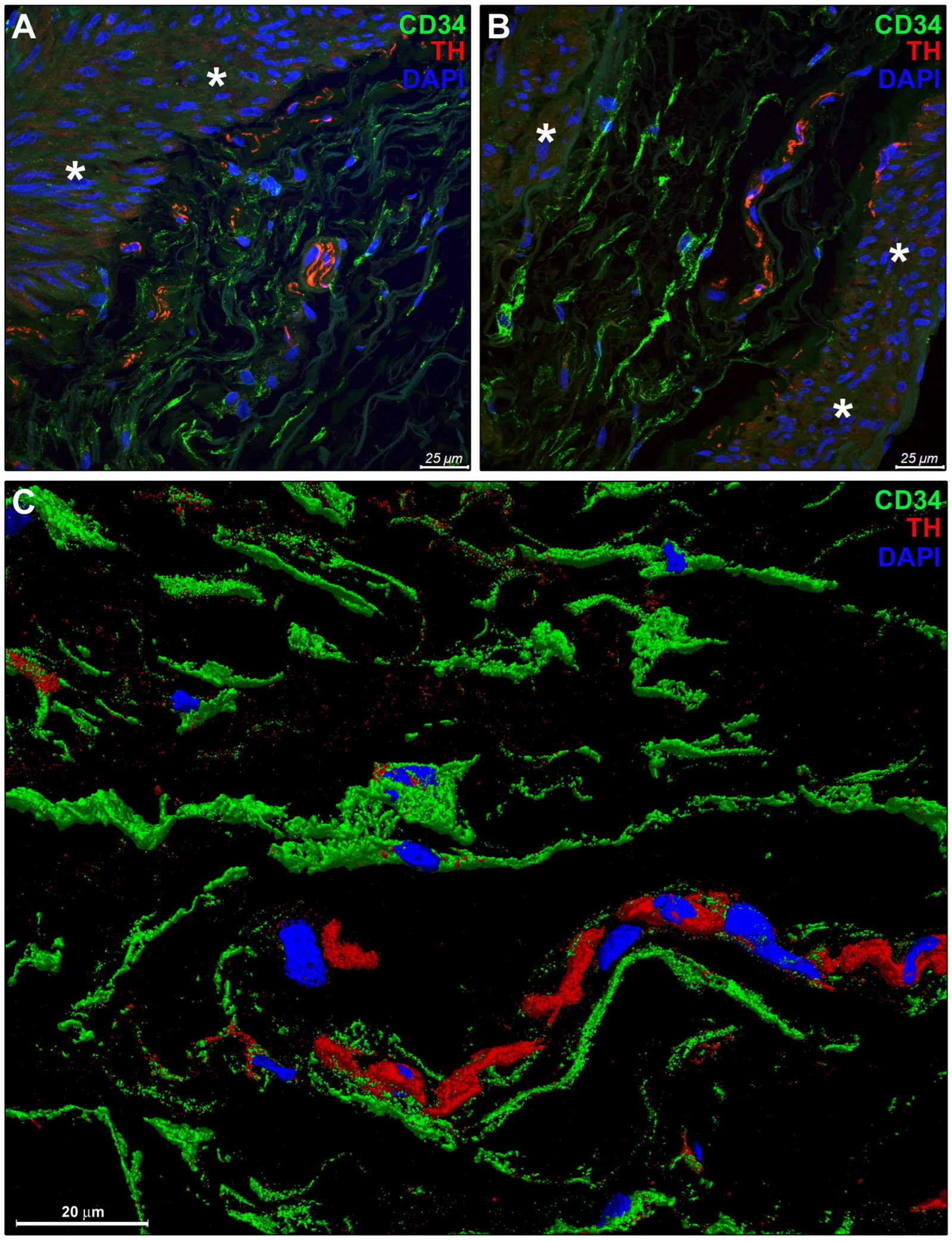
Spatial association between telocytes (TCs) and autonomic nerve fibers in the human superficial fascia (SF). (**A**,**B**) Representative 3D maximum projection confocal laser scanning microscopy images of SF sections immunostained for CD34 (green) and tyrosine hydroxylase (TH; red). In all panels, nuclei are counterstained in blue with 4′,6-diamidino-2-phenylindole (DAPI). The extensive network of CD34+ TCs is in close relationship with TH+ sympathetic nerve fibers distributed around the wall of fascial small arteries/arterioles (asterisks). (**C**) Representative 3D volume rendering of CD34/TH confocal immunofluorescence highlighting the spatial distribution of CD34+ TCs and their proximity to TH+ nerve fibers. Photomicrographs are representative of SF tissue sections from three independent biological donors. Scale bars are indicated in each panel.

To investigate the relationship between TCs and the autonomic innervation of the SF, double immunostaining for CD34 and tyrosine hydroxylase (TH) was performed (Figure 9). The networks of CD34+ TCs demonstrated close spatial association with TH+ sympathetic nerve fibers, especially in the connective tissue surrounding small arteries/arterioles (Figure 9A,B). As shown in Figure 9C, the 3D volume rendering of CD34/TH confocal immunofluorescence further highlighted the spatial distribution of the TC cytoplasmic processes and their proximity to TH+ nerve fibers.

### 2.7. Ultrastructural Validation of Telocytes in the Human Superficial Fascia

**Figure 10:**
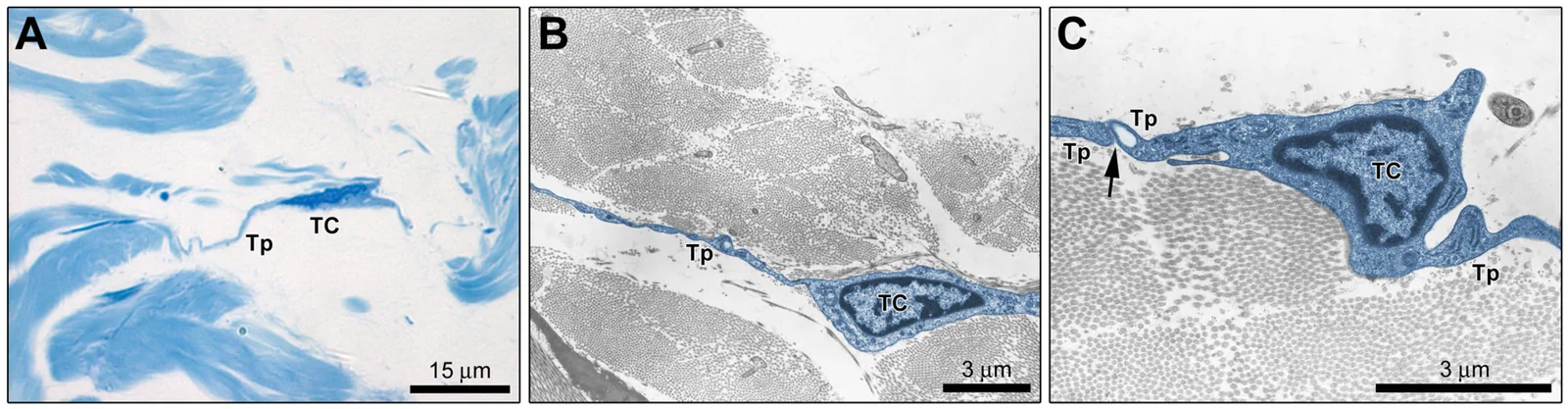
Identification of telocytes (TCs) in semithin and ultrathin sections of the human superficial fascia (SF). (**A**) Semithin section of the SF stained with toluidine blue showing a spindle-shaped TC with long processes interspersed within the fascial stroma. (**B**,**C**) Representative transmission electron microscopy images of SF ultrathin sections. TCs are characterized by a small cell body with a large nucleus and extremely long, slender telopodes (Tp) exhibiting a moniliform appearance, conferred by the alternation of thin segments (podomers) and dilated portions (podoms). In (**B**) and (**C**), TCs and telopodes have been digitally colored in blue to highlight their characteristic morphology and relationship with collagen bundles. The arrow in (**C**) indicates an intercellular contact. Electron micrographs are representative of SF tissue samples from three independent biological donors. Scale bars are indicated in each panel.

The identification of TCs in the SF was further validated by an analysis of semithin sections by light microscopy and ultrathin sections by TEM (Figure 10). Toluidine blue-stained semithin sections highlighted spindle-shaped cells with extremely long processes (Figure 10A). At the ultrastructural level, TCs were unequivocally identified by their unique morphology: a small cell body containing a large nucleus and very long, thin telopodes exhibiting the characteristic alternation of slender segments (podomers) and dilated regions (podoms) accommodating the endoplasmic reticulum and mitochondria (Figure 10B,C).

## 3. Discussion

The present morphological study provides the first evidence of TCs within the human SF, alongside their comprehensive characterization. While previous limited research has identified TCs in the deep muscular fascia [52,53,54], our findings reveal that these cells are also a prominent component of the hypodermal interstitial microenvironment. Indeed, by integrating immunohistochemical, confocal immunofluorescence, and ultrastructural analyses, we describe a TC population organized into extensive 3D networks, which likely play a key role in maintaining SF homeostasis and coordinating tissue-specific functions.

Through a series of double immunofluorescence confocal analyses combining CD34—the hallmark, though non-specific, marker defining these cells as TCs/CD34+ stromal cells [12,15,18,36,41,55]—with each target marker, we precisely delineated the immunophenotypic profile of TCs in the human SF as CD34+/PDGFRα+/LGR5+ while systematically excluding positivity for endothelial (CD31–), vascular smooth muscle/pericytic (α-SMA–), and mast cell (c-kit–) markers. Among these, a particularly significant finding of our study is the expression of LGR5, which has previously been identified as a functional marker for TCs in the intestinal mucosa, where these cells serve as a critical signaling source for maintaining the epithelial stem cell niche and regulating the epithelial spatial expression programs along the villus axis [24,56,57,58]. In the context of the SF, which is increasingly recognized as a dynamic cellular and extracellular reservoir harboring preadipocytes and mesenchymal progenitors [59,60,61], the presence of LGR5+ TCs suggests they may function as local “nursing cells”. Similar to their documented involvement in skeletal muscle, where TCs are thought to support satellite cell activation and myogenic differentiation through direct structural support and paracrine signaling [22,41], fascial TCs may participate in establishing a perivascular stem cell niche within the hypodermal interstitium. Given that the SF serves as a primary source of cells and matrix for cutaneous wound repair and tissue remodeling [2,62,63], their perivascular positioning might support a role for TCs in coordinating the survival, spatial guidance, and lineage commitment of local progenitor cells involved in fascial adipogenesis and extracellular matrix remodeling.

Beyond their putative association with progenitor microenvironments, our confocal immunofluorescence data highlight a strategic spatial arrangement of TCs around D2-40+ lymphatic capillaries and pre-collectors. This distinct anatomical distribution points to a potential involvement of TCs in the structural and physiological regulation of the fascial lymphatic network. Recent landmark in vivo studies have provided functional evidence that TCs form a specialized interstitial sensory network bridging synovial lymphatic capillaries to collecting lymphatic vessels, which is profoundly impaired in murine models of rheumatoid arthritis [48,64,65,66]. In this pathway, TCs act as mechanosensors that detect variations in interstitial fluid pressure and transduce these signals to perilymphatic mast cells; upon activation, mast cells degranulate and release mediators such as histamine, which directly stimulate smooth muscle cell contractions in collecting lymphatics to drive the clearance of joint effusions [48,64,66]. The close spatial relationship between c-kit– TCs and c-kit+ mast cells observed in our human SF specimens provides a clear morphological foundation for a similar functional TC–mast cell–lymphatic axis in the hypodermis. However, although our 3D confocal volume rendering reconstructions clearly demonstrate tight spatial proximity between TCs and both lymphatic capillaries and c-kit+ mast cells, these observations currently remain purely descriptive. Therefore, whether a similar functional TC–mast cell–lymphatic axis operates in human SF—mediating paracrine cross-talk, fluid homeostasis, or lymphangiogenesis—currently represents a plausible hypothesis that warrants dedicated functional and in vitro investigation. Nevertheless, given that the human SF hosts an extensive lymphatic plexus essential for interstitial fluid homeostasis, a potential disruption of this cellular network could impair pressure-sensing and lymphatic pumping, providing novel morphological insights into fascial fluid stasis, tissue congestion, and conditions such as lymphedema [2,5,67,68,69].

The anatomical integration of TCs within the human SF is further underlined by the close spatial relationship observed between these stromal cells and autonomic nerve fibers. The SF is one of the most densely innervated connective layers of the body, hosting a substantial population of TH+ sympathetic nerve fibers involved in thermoregulation and modulation of exteroception thresholds [2,4]. Our observations demonstrate that CD34+ TC networks extend in close proximity to TH+ sympathetic fibers and occupy strategic adventitial positions around blood vessels. This architectural arrangement points to a putative integration of TCs into the autonomic and vascular regulatory apparatus of the hypodermis. As interstitial “connecting cells”, TCs are structurally equipped to mediate local vasomotor responses and tissue homeostasis, potentially participating in rapid cellular responses to thermal variations or systemic sympathetic stress—factors known to alter biomechanical tension and vascular patency within the SF plane [2].

From a structural perspective, the SF acts as a mechanical sliding interface between the skin and the underlying musculoskeletal structures [2,3]. Of note, recent paradigm-shifting in vivo evidence has redefined the SF as a dynamic reservoir capable of mobilizing entire “tissue assemblages”—pre-assembled blocks of matrix, cells, and microvessels—to repair deep wounds [2,62,63]. In this tissue mobilization framework, it is tempting to speculate that the extensive 3D network of TCs identified here might serve as a structural organizational hub. Indeed, through their extremely long telopodes, TCs may provide an architectural scaffold that maintains spatial relationships across the fascial stroma, as previously proposed for myocardial tissue organization in developing and adult heart [14,19,44,45,70]. Moreover, while functional studies are required to confirm the precise mechanisms, established literature in other organs indicates that TCs participate in intercellular communication not only through direct homocellular and heterocellular contact junctions but also via the secretion of paracrine factors and extracellular vesicles, mainly exosomes and ectosomes [18,22,36,37,38,41,47,49,71,72,73,74,75,76]. Through these combined pathways, along with their known expression of matrix metalloproteinases that generate permissive conduits within matrix sublayers [22], TCs might help direct the migration and spatial alignment of fibroblasts, fibro-adipogenic progenitors (FAPs), and immune cells during tissue repair.

Conversely, the progressive impairment or numerical reduction in the TC network—a concept described in other tissues as “telocytopathy” [77,78,79]—could represent a common pathophysiological driver in several chronic fascial dysfunctions, which deserves thorough investigation. Evidence from other connective tissues, such as the synovium in rheumatoid arthritis and the skin in systemic sclerosis, links “telocytopathy” directly with chronic inflammation, impaired microvascular control, and fibrotic progression [66,78,80,81]. Furthermore, emerging evidence demonstrates that TCs may exert an anti-fibrotic effect by modulating the synthetic activity of dermal fibroblasts, suppressing their differentiation and phenotypic transition into α-SMA-expressing myofibroblasts via paracrine signaling and secretome components [80,82]. Likewise, within the SF, a compromise of the TC network and the consequent loss of this anti-fibrotic protection might unleash unchecked myofibroblast activation, leading to excessive collagen deposition, fibrotic remodeling, and pathological matrix stiffening characteristic of conditions such as the diabetic foot [83]. Additionally, given that the vast majority of fibrous septa involved in skin dimpling originate from the SF, “telocytopathy” within this layer might also play a role in the pathological shortening of retinacula cutis septa and localized fluid retention observed in cellulite [2,84]. Similarly, considering the close proximity of TCs to TH+ autonomic fibers, prospective investigations into TC integrity could yield valuable insights into altered thermal tolerance and soft-tissue tenderness reported in patients with fibromyalgia [2]. However, it must be emphasized that these potential disease associations remain strictly hypothesis-generating at this stage, serving as conceptual frameworks to guide future histopathological and translational clinical studies.

Notwithstanding these novel anatomical insights, some limitations of the present study must be acknowledged. First, our findings are inherently descriptive and morphological; while immunohistochemical, confocal immunofluorescence, and ultrastructural analyses consistently document the presence and spatial topography of TCs in human SF, direct functional assays were not performed. Consequently, all hypotheses regarding paracrine signaling, mechanotransduction, and interstitial fluid regulation rely on established paradigms from other tissue models and will require rigorous experimental validation through future in vitro functional assays (e.g., cell co-cultures). Second, our observations are based on a limited cohort (*n* = 3; two females and one male, aged 40–45 years) from a single anatomical site (abdominal tissue) and a narrow demographic range. Although we performed a quantitative morphometric analysis of TC density per HPF across both the SF adipose-rich and fibrous connective tissue compartments—revealing no statistically significant differences among independent biological donors—future studies on larger cohorts from diverse anatomical regions, across broader age groups, and encompassing different clinical backgrounds will be necessary to establish potential regional variations or age- and sex-dependent differences in TC distribution. Third, it is important to underline that immunohistochemical identification of TCs remains challenging due to the lack of a single, exclusive molecular marker, necessitating reliance on a multi-marker positive and negative panel [12,41,55]. Furthermore, advanced automated spatial distribution analyses (such as nearest-neighbor or distance-distribution algorithms) are currently constrained by this lack of an exclusive marker, as CD34 is co-expressed by blood capillary endothelial cells; future identification of highly specific TC markers will enable automated quantitative spatial modeling to fully map their microenvironmental distribution.

To build upon these findings, future research directions should focus on in-depth molecular and functional characterization. The implementation of single-cell RNA sequencing and spatial transcriptomics on isolated human SF cells will be essential to define the unique transcriptomic signature of fascial TCs, uncover potential intra-tissue subtypes, as previously demonstrated in other anatomical districts [37,41,85,86], and definitively differentiate them from classical adventitial fibroblasts and FAPs. Furthermore, in vitro functional assays utilizing isolated fascial TCs and/or their secretome and extracellular vesicles could elucidate their proposed paracrine effects on fascial preadipocytes, fibroblast-to-myofibroblast transition, and extracellular matrix dynamics. In addition, targeted in vivo depletion studies in appropriate animal models will be paramount to establish the causal roles of TCs in maintaining SF architecture, interstitial fluid homeostasis, and tissue repair under physiological and pathological conditions. Finally, investigating how mechanical loading, physical therapy, or specific manual interventions may influence TC density and structural integrity in the SF could establish a mechanistic foundation for novel therapeutic strategies targeting fascial pain, stiffness, and impaired wound healing.

## 4. Materials and Methods

### 4.1. Human Superficial Fascia Sample Collection

Samples of SF (~700 µm in thickness) were collected from the subumbilical region of three adult donors (Donor 1: female, 42 years; Donor 2: female, 40 years; Donor 3: male, 45 years) undergoing abdominoplasty surgery at the Plastic and Reconstructive Surgery Unit of the University of Padova Medical Center. The volunteers were overweight or obese (BMI range: 28–30 kg/m^2^), with no reported comorbidities. All procedures were conducted in accordance with the Declaration of Helsinki. The study protocol was approved by the local Ethics Committee (approval no. 6032/AO/24, approved on 16 October 2024), and written informed consent was obtained from all participants prior to enrollment. For each donor, six specimens (approximately 0.5 × 1 cm) were randomly collected from different areas of the abdominal region. The SF layers were isolated from the surrounding adipose tissue, fixed in 10% buffered formalin, dehydrated through a graded ethanol series and xylene, and embedded in paraffin wax. From each paraffin block, 5 µm thick sections were cut using a microtome, dewaxed, and rehydrated for subsequent hematoxylin and eosin staining, immunohistochemistry, and immunofluorescence.

### 4.2. Immunohistochemistry

Paraffin-embedded SF tissue sections (5 µm thick) were dewaxed in xylene, rehydrated through a graded ethanol series to water, and subjected to heat-induced epitope retrieval in a 10 mM sodium citrate buffer (pH 6.0; Sigma-Aldrich, St. Louis, MO, USA) for 10 min in a microwave oven. To inhibit endogenous peroxidase activity, slides were treated with a 3% hydrogen peroxide solution for 15 min at room temperature. Non-specific antibody binding was prevented by applying Ultra V block (catalog no. TA-125-UB; Lab Vision, Fremont, CA, USA) for 10 min. Subsequently, the samples were incubated overnight at 4 °C with a mouse monoclonal primary antibody against CD34 (dilution 1:50; catalog no. M7165; Dako/Agilent Technologies, Santa Clara, CA, USA). Negative controls (Supplementary Figure S2) were prepared by replacing the primary antibody with an irrelevant isotype-matched mouse IgG (Sigma-Aldrich). After washing in phosphate-buffered saline (PBS, pH 7.4; Euroclone, Milan, Italy), the immunoreactivity was detected by sequential 10 min incubations with biotinylated secondary antibodies (catalog no. TP-125-BN; Lab Vision) and streptavidin–peroxidase conjugate (catalog no. TS-125-HR; Lab Vision). Chromogenic development was achieved using 3-amino-9-ethylcarbazole (AEC; catalog no. TA-125-SA; Lab Vision). Sections were counterstained with Mayer’s hematoxylin (Bio-Optica, Milan, Italy), coverslipped using VectaMount AQ aqueous mounting medium (catalog no. H-5501; Vector Laboratories, Newark, CA, USA), and examined under a Leica DM4000 B optical microscope (Leica Microsystems, Mannheim, Germany). CD34+ stromal cells/TCs were counted in six randomly chosen microscopic HPFs (40× objective) of both the adipose-rich compartment and the fibrous connective tissue of the SF per section. Three sections from different paraffin-embedded tissue blocks were examined for each of the three independent biological donors. Only CD34+ spindle-shaped cells with well-defined nuclei were counted. Counting was performed by two independent observers (M.M. and I.R.), and the mean of the two different observations for each sample was used for statistical analysis.

### 4.3. Immunofluorescence and Confocal Laser Scanning Microscopy

To characterize the immunophenotype of TCs in the SF, 5 µm thick paraffin-embedded tissue sections were dewaxed, rehydrated, and subjected to heat-induced epitope retrieval in 10 mM sodium citrate buffer (pH 6.0; Sigma-Aldrich). To minimize tissue autofluorescence, slides were treated with a 2 mg/mL glycine solution in PBS (Euroclone) for 10 min at room temperature. Non-specific binding sites were blocked by incubating the sections for 1 h with 1% bovine serum albumin (BSA; Sigma-Aldrich) in PBS. Tissue sections were then incubated overnight at 4 °C with primary antibody cocktails containing combinations of mouse, rabbit, or goat antibodies (listed in Table 1). Irrelevant isotype-matched and concentration-matched IgGs in place of primary antibodies served as negative controls (Supplementary Figure S2). The following day, sections were incubated for 1 h at room temperature in the dark with fluorophore-conjugated secondary antibodies (diluted 1:200 in PBS containing 1% BSA), including Alexa Fluor 488-conjugated donkey anti-mouse IgG, Rhodamine Red-X-conjugated goat anti-rabbit IgG, or Alexa Fluor 568-conjugated donkey anti-goat IgG (all from Invitrogen, Carlsbad, CA, USA). Nuclei were counterstained with 4′,6-diamidino-2-phenylindole (DAPI; Chemicon International, Merck Millipore, Billerica, MA, USA). Slides were mounted using Fluoro-Gel mounting medium (catalog no. 17985-10; Electron Microscopy Sciences, Hatfield, PA, USA). Fluorescence imaging was performed using a Leica DM4000 B optical microscope or a Leica Stellaris 5 confocal laser scanning microscope (Leica Microsystems) equipped with a Plan-Apochromat 63×/1.40 NA oil immersion objective. To analyze the 3D organization of the stromal networks, Z-stacks consisting of 16 optical sections with a 300 nm step size were acquired and processed to generate maximum intensity projection images. Maximum projection confocal laser scanning microscopy images were subjected to 3D volume rendering with the Leica Application Suite X (LAS X) software (Leica Microsystems).

### 4.4. Transmission Electron Microscopy

Ten small specimens from each of the collected SF samples were randomly selected and immediately fixed in 2.5% glutaraldehyde (Agar Scientific Elektron Technology, Stansted, UK) in 0.1 M phosphate buffer, post-fixed in 1% osmium tetroxide (Agar Scientific Elektron Technology) in 0.1 M phosphate buffer, dehydrated in a graded ethanol series, and then embedded in an Epoxy Embedding Medium Kit (Sigma-Aldrich). Semithin (0.5 µm thick) and ultrathin (60 nm thick) sections were cut with an RMC PowerTome ultramicrotome (Boeckeler Instruments, Tucson, AZ, USA). Semithin sections were stained with 1% toluidine blue on a hot plate, then observed and photographed using a Leica DMR microscope (Leica Microsystems). Ultrathin sections were collected on 300-mesh copper grids and counterstained with an alcoholic solution of 1% uranyl acetate, followed by a lead solution. Specimens were observed and photographed using a Hitachi H-300 Transmission Electron Microscope (Hitachi, Tokyo, Japan).

### 4.5. Statistical Analysis

Data were analyzed using GraphPad Prism 5 software. Data normality was verified with the Shapiro–Wilk test. Differences in the number of TCs/HPF across the three independent biological donors were assessed using one-way ANOVA with Tukey’s post hoc adjustment. Values are expressed as mean ± standard deviation (SD), and a threshold of *p* < 0.05 was chosen to denote statistical significance.

## 5. Conclusions

This study provides the first morphological, immunophenotypic, 3D confocal volume rendering, and ultrastructural demonstration of TCs within the SF. Organized into extensive 3D networks across both adipose-rich and fibrous compartments, fascial TCs display strategic spatial associations with lymphatic capillaries, mast cells, autonomic nerve fibers, and the perivascular microenvironment. These findings significantly expand our current understanding of hypodermal anatomy, positioning TCs as potential structural organizational hubs that may be involved in interstitial fluid balance, autonomic-vascular integration, and the regulation of local progenitor cell niches. Furthermore, the identification of this specialized cellular network offers a novel morphological framework to explore the pathogenesis of fascial dysfunctions, wherein the loss of TC structural support—alongside their hypothesized role in anti-fibrotic signaling and myofibroblast regulation—may drive pathological tissue stiffening and fibrotic remodeling. Future in vitro, functional, and transcriptomic investigations will be essential to decipher the precise molecular signatures and functional cross-talk of fascial TCs, evaluating their potential as therapeutic targets in clinical conditions such as lymphedema, diabetic fascial stiffening, and chronic soft-tissue pain.

## Figures and Tables

**Table 1:** List of primary antibodies, their suppliers, and working dilutions.

Antibody	Host Species	Catalog No.	Supplier	Dilution
anti-CD34	mouse	M7165	Dako/Agilent Technologies, Santa Clara, CA, USA	1:50 (IHC, IF) †
anti-CD34	rabbit	ab81289	Abcam, Cambridge, UK	1:100 (IF) ‡
anti-CD31/PECAM-1	rabbit	ab28364	Abcam, Cambridge, UK	1:50 (IF)
anti-α-SMA	rabbit	ab5694	Abcam, Cambridge, UK	1:100 (IF)
anti-PDPN (D2-40)	mouse	M3619	Dako/Agilent Technologies, Santa Clara, CA, USA	1:50 (IF)
anti-c-kit/CD117	rabbit	A4502	Dako/Agilent Technologies, Santa Clara, CA, USA	1:100 (IF)
anti-PDGFRα	goat	AF-307-NA	R&D Systems, Minneapolis, MN, USA	1:100 (IF)
anti-LGR5	rabbit	PA5-87974	Thermo Fisher Scientific, Waltham, MA, USA	1:100 (IF)
anti-TH	rabbit	GTX113016	GeneTex, Irvine, CA, USA	1:100 (IF)

† Used for CD34/CD31, CD34/α-SMA, CD34/c-kit, CD34/PDGFRα, CD34/LGR5, and CD34/TH double immunofluorescence. ‡ Used for CD34/D2-40 double immunofluorescence. PECAM-1, platelet-endothelial cell adhesion molecule-1; α-SMA, α-smooth muscle actin; PDPN, podoplanin; PDGFRα, platelet-derived growth factor receptor α; LGR5, leucine-rich repeat-containing G-protein-coupled receptor 5; TH, tyrosine hydroxylase; IHC, immunohistochemistry; IF, immunofluorescence.

## Data Availability

The original contributions presented in this study are included in the article. Further inquiries can be directed to the corresponding author.

## Supplementary Materials

The following supporting information can be downloaded at https://www.mdpi.com/article/10.3390/ijms27209331/s1.

## Abbreviations

The following abbreviations are used in this manuscript:

SF	Superficial fascia
TC	Telocyte
TEM	Transmission electron microscopy
3D	Three-dimensional
HPF	High-power field
PECAM-1	Platelet-endothelial cell adhesion molecule-1
α-SMA	α-smooth muscle actin α-SMA
PDGFRα	Platelet-derived growth factor receptor α
LGR5	Leucine-rich repeat-containing G-protein-coupled receptor 5
TH	Tyrosine hydroxylase
FAPs	Fibro-adipogenic progenitors
SD	Standard deviation
