# Supplementary material for: Unveiling Telocyte Networks in the Human Superficial Fascia: A New Component of the Hypodermal Interstitial Microenvironment
Source: Int J Mol Sci. 2026 Oct 20;27(20):9331. doi: 10.3390/ijms27209331 (PMC13705334; doi:10.3390/ijms27209331)
Supplement: Supplementary file 1 [file ijms-27-09331-s001.zip › Supplementary Figure S1.pdf]

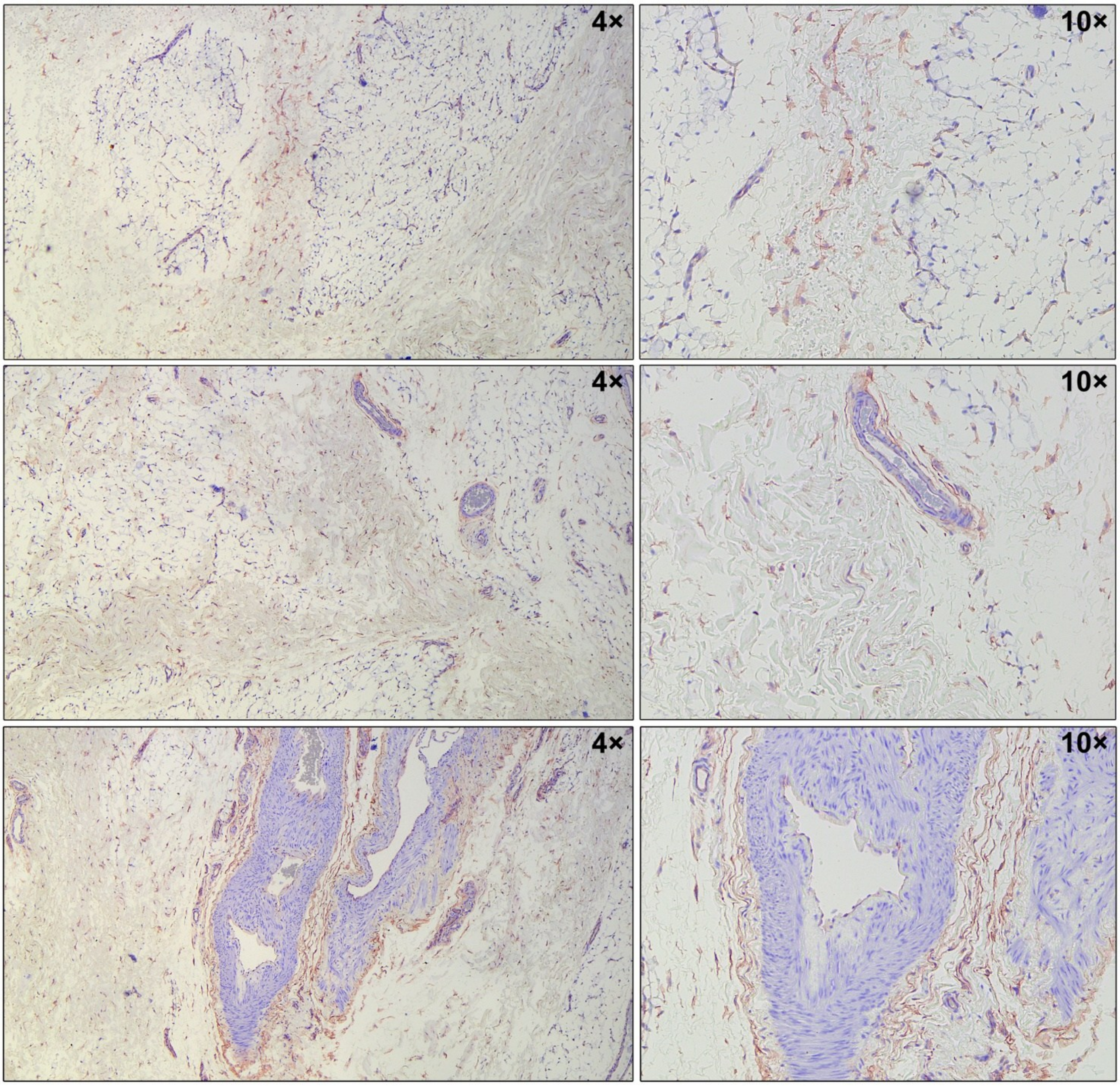

Figure S1. Representative photomicrographs of human superficial fascia sections subjected to CD34 immunohistochemistry and counterstained with hematoxylin. Images on the left and right were acquired with the 4× and 10× objectives, respectively. CD34+ stromal cells/telocytes (brownish-red) appear widely distributed throughout both the adipose-rich compartment and the fibrous connective tissue of the superficial fascia. The lower panels show extensive networks of CD34+ stromal cells/telocytes around small arteries/arterioles. Photomicrographs are representative of tissue sections obtained from three independent biological donors.
