# Supplementary material for: Unveiling Telocyte Networks in the Human Superficial Fascia: A New Component of the Hypodermal Interstitial Microenvironment
Source: Int J Mol Sci. 2026 Oct 20;27(20):9331. doi: 10.3390/ijms27209331 (PMC13705334; doi:10.3390/ijms27209331)
Supplement: Supplementary file 1 [file ijms-27-09331-s001.zip › Supplementary Figure S2.pdf]

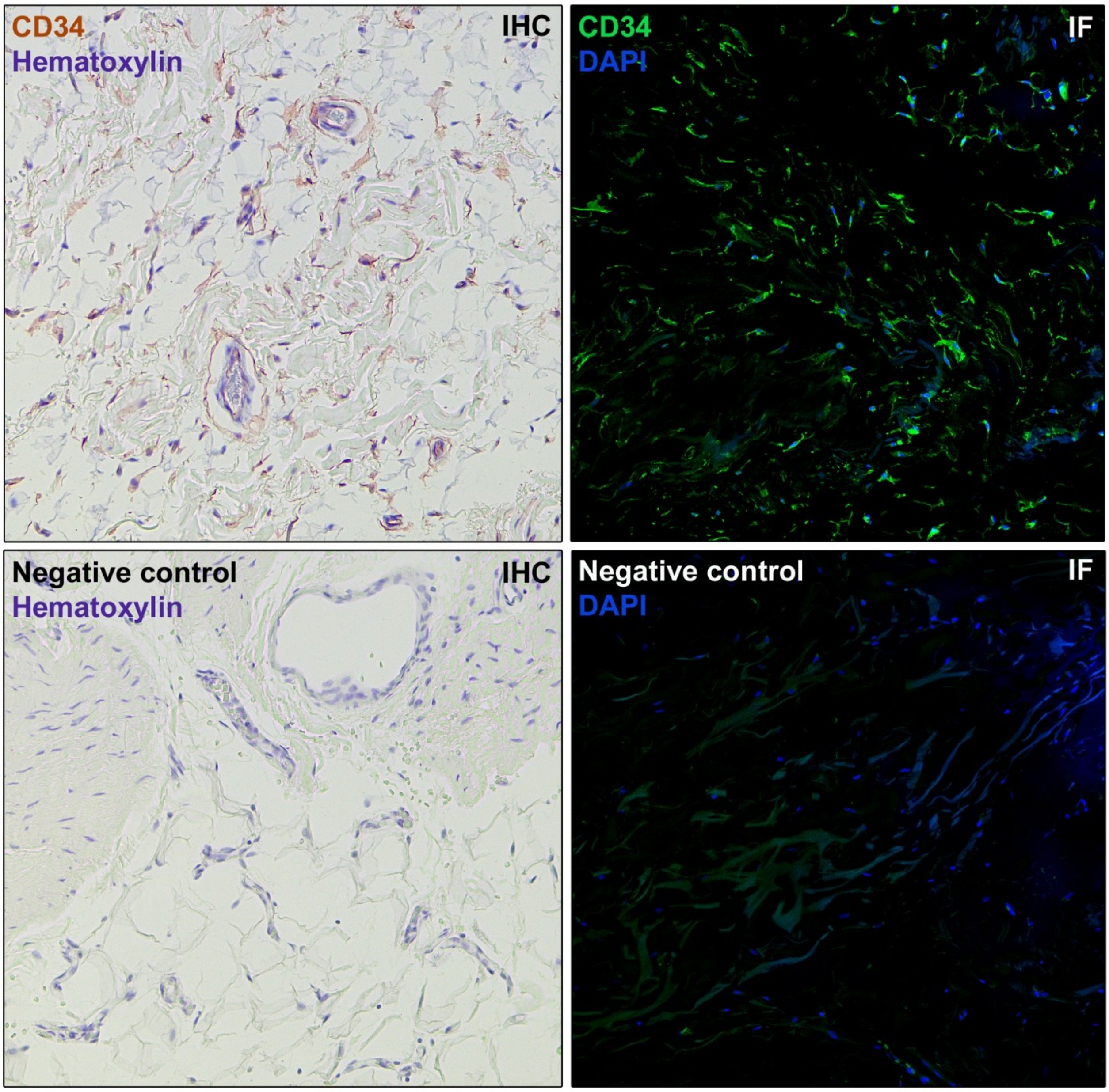

Figure S2. Representative photomicrographs of negative controls for immunohistochemistry and immunofluorescence. Upper panels show positive immunostaining in human superficial fascia sections incubated with a primary anti-CD34 antibody. Lower panels show negative controls obtained by incubating tissue sections with irrelevant isotype-matched and concentration-matched IgG at the same magnification, confirming the absence of non-specific signal or background fluorescence. Nuclei are counterstained with hematoxylin for immunohistochemistry and 4′,6-diamidino-2-phenylindole (DAPI) for immunofluorescence. Photomicrographs are representative of tissue sections obtained from three independent biological donors. IHC, immunohistochemistry; IF, immunofluorescence.
